# Have the Locality and Surroundings an Influence upon the Recurrence of Malignant Disease after Operation?

**Published:** 1903-07-25

**Authors:** D'Arcy Power

**Affiliations:** Senior Surgeon to the Victoria Hospital for Children, Chelsea; Assistant Surgeon to St. Bartholomew's Hospital, E.C.


### Hospital Clinics and Medical Progress.

### HAVE THE LOCALITY AND SURROUNDINGS AN INFLUENCE UPON THE RECURRENCE OF MALIGNANT DISEASE AFTER OPERATION?

Abstract of a Paper by D'Arcy Power, F.R.C.S.-Eng., Senior Surgeon to the Victoria Hospital for Children, Chelsea; Assistant Surgeon to St. Bartholomew's Hospital, E.C.

Mr. D'Arcy Power read a paper at the Liverpool Congress of Public Health in which he said that the aberrant course run by malignant growths of apparently identical structure was a matter of common knowledge, but he thought that no one had yet made any attempt to determine whether the irregularities were due to the malignant growth, to the individual in whom it grew, or to the surroundings of the individual. It seemed to be extremely desirable to ascertain whether the recurrence in malignant disease was really as capricious as was generally supposed or whether there might not be some underlying reason to explain why the disease reappeared in some persons but not in others, and at such varying times after the operation. He brought forward a series of cases which raised a suspicion that recurrence in cancer was not so arbitrary as it seemed to be at first sight, but might be due to such underlying causes as locality and alterations in the bodily condition of the patient. These underlying causes might accelerate, retard, or even entirely prevent the recurrence of carcinoma when the primary growth had been removed by a surgical operation. He asked, therefore, whether after an operation for malignant disease it was advisable or inadvisable to send a patient back to his old house and to his former surroundings. He thought that grief, mental worry, and a return to the original home sometimes appeared to increase the tendency to recurrence or metastatic growths after an apparently successful operation for cancer. He pointed out that a clear distinction was to be made between cases of immediate and remote recurrence after operation. Immediate recurrence being due to imperfect removal of the disease, either because the operation had been insufficient or because dissemination had taken place before it was performed. Recurrence taking place after a long interval was more likely to be a re-infection, or at any rate, a fresh attack of malignant disease, though it might reasonably be argued, on the analogy of malaria, that cancer and sarcoma sometimes remained dormant for long periods of time. Mr. Power asked for information about both kinds of recurrence. In the first place, where there were further signs of malignant disease within a year of an operation performed by a competent surgeon in an early case and upon a part where it might reasonably be expected that the entire disease would have been removed, as in a scirrhous cancer of the breast with only a moderate degree of infection of the axillary glands. Secondly, where under similar conditions recurrence or metastatic deposits had occurred after an interval of three or more years of apparent recovery. He wished to know more especially whether there had been any change of habitation or habits, or whether, from any cause, there had been influences at work which would tend to lower the general vitality of the patient. He thought it would be especially valuable to compare such cases with others, when they were comparable, in which there had been no such recurrence or metastatic deposit, either immediate or remote.

